# E-selectin-targeting lipid nanoparticles improve therapeutic efficacy and reduce side effects of bortezomib in multiple myeloma

**DOI:** 10.1038/s41408-023-00828-4

**Published:** 2023-04-07

**Authors:** Mina Maksimos, Barbara Muz, John L. Magnani, Abdel Kareem Azab

**Affiliations:** 1 Department of Radiation Oncology, Cancer Biology Division, Washington University in St. Louis School of Medicine, St. Louis, MO USA; 2 Institute of Microbiology, Friedrich Schiller University, Jena, Germany; 3 GlycoMimetics Inc., Rockville, MD USA; 4 Department of Biomedical Engineering, The University of Texas Southwestern Medical Center, Dallas, TX USA

**Keywords:** Cancer microenvironment, Targeted therapies, Drug development

**Dear Editor**,

Multiple myeloma (MM) is the second most common hematological malignancy; in 2022, the National Cancer Institute indicated about 34,470 new cases were diagnosed with MM, and about 12,640 deaths are estimated in the United States, with a 57.9% 5-year survival rate [1]. Understanding molecular mechanisms of the cell-signaling pathway in MM cells and the interaction of MM cells with their bone marrow (BM) microenvironment has led to the development of novel therapies [2]. Despite introducing novel therapies, more than 90% of MM patients relapse due to drug resistance [3]. The BM microenvironment plays a crucial role in the development of resistance to proteasome inhibitors (PIs) in MM, in which direct and indirect interaction of MM cells with the BM microenvironment induced resistance to PIs in MM [4].

The main limitation that hampers the treatment with PIs is the lack of specificity of the pharmacological effect and therefore causing dose-limiting off-target side effects (such as peripheral neuropathy and hematological toxicities). Nanoparticle delivery systems were shown to be capable of targeting large doses of therapy into the target area while sparing healthy tissues, overcoming the limitations of traditional therapies [5]. We have previously shown that delivery of PIs, such as bortezomib (BTZ), in CD38-targeted cross-linked-chitosan nanoparticles significantly reduced the toxicity profile of BTZ in vivo; however, the limited improvement of the therapeutic efficacy is most likely due to the endothelial cell barrier [6]. More recently, we have developed tumor-associated endothelial cells (TAECs)-targeted nanoparticles, which improve efficacy and reduce the side effects of bortezomib in multiple myeloma [7]. These lipid nanoparticles specifically targeted TAECs rather than targeting tumor antigens. This strategy showed that TAECs could be seen and used as a specific target for nanoparticle-based therapies rather than a barrier that limits nanoparticle delivery [7]. We have previously shown that inhibiting the interaction between MM cells and the BM microenvironment, especially endothelial cells (ECs) [4, 8–11], using a pan-selectin inhibitor GMI-1070 enhances the sensitization of MM cells to bortezomib in vitro and in vivo [4]. Similarly, we demonstrated that E-selectin inhibition reduced tumor dissemination and enhanced the efficacy of both lenalidomide and carfilzomib in MM [4, 8, 9]. This nominates E-selectin as a potential antigen in TAECs in MM, which can be a potential target for nanoparticle-based delivery of PIs in MM, for improved treatment specificity.

In this study, we hypothesized that E-selectin is a unique antigen with high expression in TAECs in MM, and that incorporating E-selectin targeting moiety GMI-1930 (Lipo E-X) to bortezomib-loaded lipid nanoparticles will improve the specificity and efficacy and reduce the side effect of bortezomib.

To assess the feasibility of our hypothesis, we first investigated the expression of E-selectin on ECs cultured alone or in co-culture with MM1.S (MM cell line). We found that ECs had a low basal expression of E-selectin; however, the expression of E-selectin was robustly increased by more than two fold due to co-cultured with MM cells (Fig. 1A). This validated our hypothesis that E-selectin is a unique antigen on TAECs in MM.

**Fig. 1:**
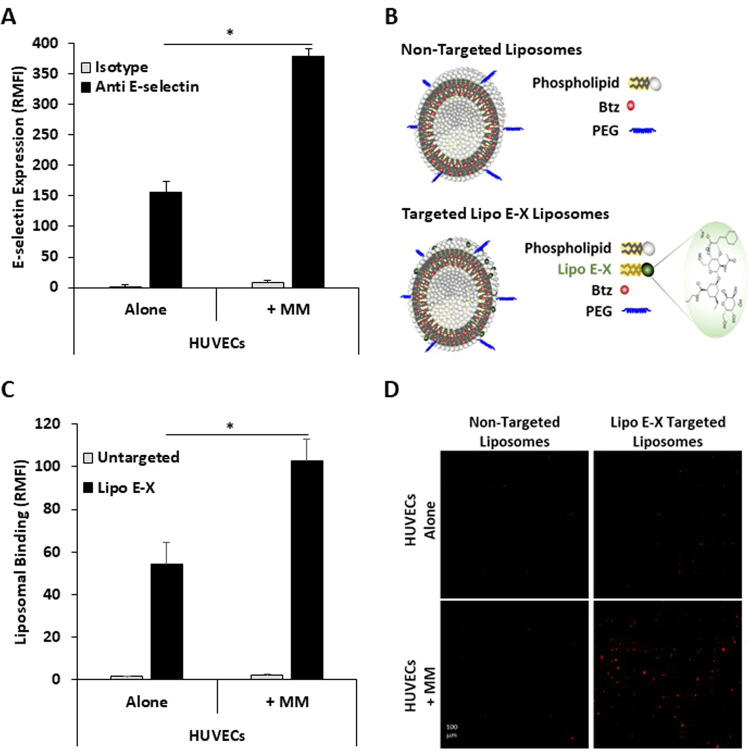
E-selectin is highly expressed on tumor-associated endothelial cells in MM, which was specifically targeted by E-selectin-targeting Lipo E-X lipid nanoparticles in vitro. E-selectin expression on ECs alone or ECs co-cultured with MM1.S cells using the 3D-Tissue Engineered Bone Marrow (3DTEBM) model (**A**). A schematic of non-targeted and Lipo E-X targeted lipid nanoparticles (**B**). Quantitative analysis of the binding of DiD-labeled non-targeted and Lipo E-X targeted lipid nanoparticles to ECs cultured with and without MM1.S in the 3DTEBM, demonstrated as RMFI of DiD detected using flow cytometry (**C**) or qualitative analysis by using Spark® Cyto imaging system (**D**). We used an unpaired Student’s *t*-test (*p < 0.05) for statistical significance.

Accordingly, we developed lipid nanoparticles targeted to E-selectin on TAECs in MM by incorporating GMI-1930, an E-selectin targeting molecule, into the lipid-bilayer of the lipid nanoparticles (Lipo E-X), and we used the same composition of the lipid nanoparticles without GMI-1930, as a non-targeted control (Fig. 1B). To evaluate the binding of the lipid nanoparticles to the TAECs, we used a 3D tissue engineered bone marrow (3DTEBM) culture model [12], in which ECs were cultured alone or in co-culture with MM1.S, as previously described [7]. Cultures were treated with DiD-fluorescently labeled Lipo E-X targeted and non-targeted lipid nanoparticles, and the level of binding of lipid nanoparticles to ECs was evaluated qualitatively using Spark®Cyto imaging system, or the cultures were digested, and binding was evaluated quantitatively using flow cytometry. Quantitatively, the binding of the non-targeted lipid nanoparticles to ECs was low when cultured alone or when co-cultured with MM. In contrast, Lipo E-X targeted lipid nanoparticles had higher binding to EC alone, due to basal E-selectin expression; and the binding doubled (two fold) when ECs were co-cultured with MM, due to E-selectin over expression (Fig. 1C). Qualitatively, fluorescence imaging of the cultures confirmed the binding patterns observed by flow cytometry (Fig. 1D). Together, Fig. 1 implies that the TAECs in MM have a high and specific expression of E-selectin, which was successfully targeted by lipid nanoparticles with E-selectin binding molecule GMI-1930 (Lipo E-X) in vitro.

We then examined the biodistribution of E-selectin-targeting Lipo E-X and non-targeted lipid nanoparticles in vivo. We injected the lipid nanoparticles intravenously (iv) into MM-bearing NCG mice (Charles River, Wilmington, MA) previously inoculated with 2 × 10^6^ MM1.S-GFP-Luc cell/mouse, and MM progression in mice was confirmed by bioluminescent imaging (BLI). Twenty-four hours post nanoparticles injection, mice were sacrificed and their BM, kidney, and liver were harvested. Then, we analyzed the binding of the lipid nanoparticles to mononuclear cells (MNCs) and quantified the levels of MM-GFP+ cells, in each organ. We found a significantly higher accumulation of the Lipo E-X lipid nanoparticles in the BM and kidney, compared to the non-targeted lipid nanoparticles (Fig. 2A). We also found a high non-specific accumulation of both targeted and non-targeted particles in the liver. Liver accumulation is expected, as lipid nanoparticles are known to accumulate in the liver due to their lipidic nature and their capture by Kupffer cells. Although BTZ-loaded lipid nanoparticles may have side effect in the lever; however, we expect these to be mild due to the lower activity of proteasomes in the liver compared to MM cells. Moreover, we examined the correlation between the presence of MM in the organs and the specificity of the accumulation of lipid nanoparticles; we determined the specific accumulation as the ratio of the binding of targeted / non-targeted lipid nanoparticles and the presence of MM was expressed as the % of MM-GFP+ in MNCs in each organ. We found a direct linear correlation between the presence of MM1 cells and the specific accumulation of targeted lipid nanoparticles in each organ (Fig. 2B). Each quantified value is presented as mean ± standard deviation from four biological replicates, and we used an unpaired Student’s t-test (*p < 0.05) for statistical significance.

**Fig. 2:**
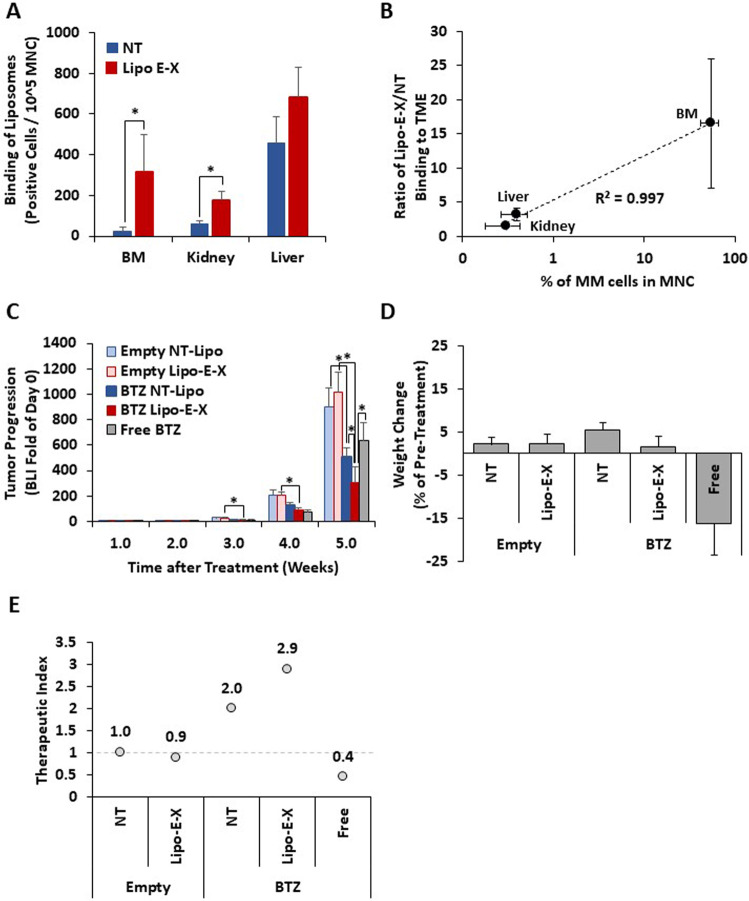
E-selectin-targeting Lipo E-X lipid nanoparticles specifically accumulate in the BM of MM-bearing mice and improve the efficacy and reduce the side effects of BTZ in vivo. **A** MM.1S-GFP-Luc cells (2 × 10^6^ cells per mouse) were injected intravenously (IV) into immunodeficient mice (*n* = 4, NCG mice, 8-week-old, Charles River Laboratories). The disseminated tumor developed over the next four weeks, which was confirmed using BLI. Pre-labeled non-targeted liposomes (DiI) and E-Selectin-targeted Lipo-E-X liposomes (DiD) were mixed in a 1:1 ratio and injected IV at 1 mg/100uL liposomes per mouse. For the biodistribution and binding efficacy determination of these two formulations, mice were sacrificed 24 h post-liposome injection. BM, kidney, and liver were harvested, washed, filtered, run using flow cytometry, and analyzed for the mean fluorescence intensity (MFI) of DiI and DiD in MM-GFP+ cells, as well as in GFP- population, i.e., tumor microenvironment using the FlowJo® software. Biodistribution of fluorescently labeled non-targeted and Lipo E-X targeted lipid (number of positive cells per 10^5^ of MNCs) in different organs in MM-bearing mice, was determined 24 h following a single iv injection. **B** The correlation between the ratio of Lipo E-X/non-targeted lipid nanoparticles binding and the presence of MM-GFP+ cells (% of MNCs) in different organs. **C** MM.1S-GFP-Luc cells were injected intravenously into immunocompromised NCG mice (*n* = 40). Tumor progression was confirmed using BLI at two weeks post-injection, and (5) BTZ as a free drug. Then mice were randomized into 5 groups of 8 mice each and treated once a week with: (1) empty non-targeted liposomes, (2) empty Lipo E-X liposomes, (3) BTZ-loaded non-targeted liposomes, and (4) BTZ-loaded Lipo E-X liposomes. All liposomal formulations (empty or loaded) were given at 10 mg/kg of liposomes, and BTZ-treated groups (free or liposomal) were given at an equivalent dose of 1 mg/kg. Tumor progression was assessed weekly by BLI and normalized to day 0 (initiation of post-treatment). **D** Percentage of weight change of mice in each group at week 5 normalized to their weight pre-treatment. **E** The value of the therapeutic index of different formulations of bortezomib in vivo. Each quantified value is presented as mean ± standard deviation from four biological replicates, and we used an unpaired Student’s t-test (*p < 0.05) for statistical significance.

BTZ is a gold-standard treatment for MM; however, its efficacy is limited by off-target dose-limiting side effects. Therefore, we loaded BTZ into the highly specific Lipo E-X lipid nanoparticles, as previously described [7], and tested its effect on tumor progression and side effects in vivo. Immunocompromised NCG mice were inoculated with MM.1S-GFP-Luc cells and tumor engraftment confirmation at two weeks using BLI. Mice were then randomized into five treatment groups that received weekly iv injections of (i) empty non-targeted lipid nanoparticles, (ii) empty Lipo E-X targeted lipid nanoparticles, (iii) BTZ-loaded non-targeted lipid nanoparticles, (iv) BTZ-loaded Lipo E-X targeted lipid nanoparticles, and (v) BTZ as a free drug. Mice were imaged using BLI weekly for tumor progression, and their weight was monitored daily. We found that the groups treated with control empty non-targeted, and Lipo E-X targeted formulations showed fast and comparable tumor growth. When BTZ was administered as a free drug or formulated in non-targeted lipid nanoparticles, both induced similar reduction of tumor growth by about 50% compared to empty formulations. On contrast, BTZ-loaded Lipo E-X lipid nanoparticles reduced tumor progression by 70% compared to empty formulations and significantly lower than the free drug and non-targeted formulations (Fig. 2C). Weight loss was previously described as the most common off-target side effects of BTZ in mice [13], confirmed by our group [7, 14], due to painful neuropathy that limits the mice’s movement and their ability to reach food [15]. Weight loss was observed when BTZ was delivered as a free drug, in which it induced severe weight loss in the animals (16%), while no weight loss or a slight weight gain was observed in the lipid nanoparticle groups (empty or loaded with BTZ) (Fig. 2D). Then, we developed a “therapeutic index -TI” calculation to demonstrate both the increase of therapeutic efficacy and the decrease of side effects; we defined this index as (safety score/efficacy score). The “efficacy score” was determined as the normalized BLI of each group to the empty-non-targeted nanoparticles group, and the “toxicity score” was defined based on the criteria to sac animals, in which a loss of 25% of the initial animal weight was considered a score of 0 and weight changes were subtracted from this value and normalized to empty-non-targeted nanoparticles group. Therefore, the empty-non-targeted nanoparticles group gets a TI of 1/1 = 1, because both toxicities and efficacy scores were normalized to this group. Due to the lack of therapeutic efficacy and side effects, the TI of the empty-Lipo-E-X targeted liposomes showed negligible change. BTZ formulations showed changes in TI, in which the free-drug BTZ showed low TI (0.4) due to high toxicity and moderate efficacy. Non-targeted liposomal BTZ showed moderately increased TI (1.9), reflecting the lack of side effects and moderate improvement of efficacy, mostly due to enhanced permeabilization and retention effect (EPR). Importantly, Lipo-E-X targeted liposomal BTZ showed the highest TI (2.9), reflecting the lack of side effects and significant increase in efficacy, due to EPR and active targeting to E-selectin on TAECs in the MM tumors (Fig. 2E).

In conclusion, we demonstrated that E-selectin is specifically and significantly upregulated in TAECs in MM, and that it can be used as a specific target in the MM TME. Hence, we developed E-selecting/TAECs/TME-targeting lipid nanoparticles (Lipo E-X), which improved therapeutic efficacy and reduced the off-target side effects of BTZ and enhanced its therapeutic index, as a potential solution to the dose-limiting side effects observed clinically and experimentally when using BTZ as a free drug.

## Data Availability

Raw data can be available upon request.
